# Downregulation of microRNA-29b by DNMT3B decelerates chondrocyte apoptosis and the progression of osteoarthritis via PTHLH/CDK4/RUNX2 axis

**DOI:** 10.18632/aging.103778

**Published:** 2020-11-07

**Authors:** Pengcheng Dou, Yu He, Bo Yu, Juan Duan

**Affiliations:** 1 Department of Orthopedics, The Second Xiangya Hospital, Central South University, Changsha 410011, P.R. China; 2 Department of Radiology, The Second Xiangya Hospital, Central South University, Changsha 410011, P.R. China; 3 Department of Critical Care Medicine, The Second Xiangya Hospital, Central South University, Changsha 410011, P.R. China; 4 Department of Geriatrics, The Second Xiangya Hospital, Central South University, Changsha 410011, P.R. China

**Keywords:** osteoarthritis, DNA methylation, DNMT3B, microRNA-29b, PTHLH

## Abstract

The correlation between DNA methyltransferases (DNMTs) and microRNAs (miRNAs) has been well-established, but its interaction in osteoarthritis (OA) has been barely clarified. This study aimed to analyze the relationship between DNMT3B and miR-29b as well as their implications in OA. Our results revealed that DNMT3B was downregulated while miR-29b was upregulated in OA cartilage tissues relative to normal cartilage tissues. Hypermethylation of specific CpG sites in the miR-29b promoter region induced by DNMT3B contributed to downregulation of miR-29b in OA chondrocytes. Furthermore, luciferase activity determination demonstrated that miR-29b targeted and negatively regulated the parathyroid hormone-like hormone (PTHLH). Moreover, the PTHLH upregulation induced by miR-29b methylation led to the enhancement of chondrocyte growth and suppression of their apoptosis and extracellular matrix degradation, which was achieved by the upregulation cyclin-dependent kinase 4 (CDK4) expression. Co-IP suggested that CDK4 induced ubiquitination of RUNX2, which could be enhanced by DNMT3B. In the OA mouse model induced by destabilization of the medial meniscus, overexpression of DNMT3B was observed to downregulate the expression of RUNX2 whereby preventing OA-induced loss of chondrocytes. Hence, the DNMT3B/miR-29b/PTHLH/CDK4/RUNX2 axis was found to be involved in the apoptosis of chondrocytes induced by OA, highlighting a novel mechanism responsible for OA progression.

## INTRODUCTION

Osteoarthritis (OA) is a joint disorder and a major cause of disability in a global range, characterized by progressive chondrocyte hypertrophy and apoptosis, subchondral bone sclerosis, osteophyte formation, and increase in periarticular bone remodeling [1]. Moreover, it contributes to the high global prevalence, and the lifetime risk of symptomatic knee OA is 44.7% (the most vulnerable group are elderly people) [2]. Then, potential risk factors for OA include age, gender, joint injury history, obesity, genetic factors, as well as mechanical factors such as malalignment and abnormal joint shape [3]. During the development of OA, extracellular matrix (ECM) of cartilage is actively remodeled by chondrocytes under inflammatory conditions, which results in alteration of the biomechanical environment of chondrocytes and accelerates the progression of OA [4].

Recently, the expression profiles of 723 human microRNAs (miRNAs) have been identified from normal and OA chondrocytes, which may have important diagnostic and therapeutic potential in the context of OA [5]. For instance, miR-29b has been reported to be upregulated in the dedifferentiated OA chondrocytes that contribute to collagen imbalance related to an aberrant chondrocyte phenotype [6]. Interestingly, DNA methyltransferase 3B (DNMT3B) has been reported to participate in cartilage homeostasis as well as the onset and the progression of OA and can partially rescue the osteoarthritic phenotype as a putative therapeutic target [7]. Additionally, the interaction between miR-29b and DNMT3A contributes to DNA methylation-induced silencing of miR-29b [8]. Accordingly, it has been indicated that miR-29b-3p facilitates the chondrocyte apoptosis and OA development by targeting Progranulin [9]. Moreover, articular chondrocytes exhibit activated transcription factors such as parathyroid hormone-like hormone (PTHLH), that play an inhibitory role in terminal chondrocyte differentiation and endochondral bone formation [10]. In this study, the PTHLH was predicted to be a potential target of miR-29b. Thus, we anticipated that DNMT3B might mediate the methylation of miR-29b and its target gene PTHLH to affect the OA progression.

Moreover, a previously reported study has demonstrated a positive correlation between PTHLH and cyclin-dependent kinase 4 (CDK4) in oral squamous cell carcinoma cells [11], which is vital for the substantial replicative potential of human chondrocytes *in vitro* [12]. More importantly, CDK4 can induce the degradation of the runt-related gene 2 (RUNX2) in a ubiquitination-proteasome-dependent manner, while RUNX2 is a domain transcription factor participating in the activation of genes encoding chondrocytes-specific proteins [13]. Therefore, the present study aimed at exploring the biological roles of DNMT3B in OA and its underlying mechanism associated with the miR-29b/PTHLH/CDK4/RUNX2 axis. Moreover, we also studied the therapeutic role of DNMT3B in the destabilization of the medial meniscus (DMM)-induced mouse OA model.

## RESULTS

### A negative correlation between DNMT3B and miR-29b expression was identified in patients with OA

**Figure 1:**
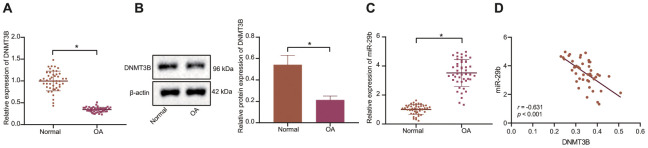
**The inverse correlation between DNMT3B and miR-29b expression is revealed in patients with OA.** (**A**) The mRNA expression of DNMT3B in normal cartilage tissues and OA cartilage tissues determined by RT-qPCR (normal, n = 46, OA, n = 46). (**B**) The protein expression of DNMT3B in tissues of five normal cartilages and five OA cartilages normalized to β-actin measured by Western blot analysis. (**C**) The expression of miR-29b in tissues of normal cartilages and OA cartilages determined by RT-qPCR (normal, n = 46, OA, n = 46). (**D**) Pearson’s analysis of the correlation between DNMT3B and miR-29b expression. ^*^ *p* < 0.05 *vs.* normal cartilages. Statistical data were measurement data and described as the mean ± standard deviation. The independent sample *t*-test was conducted for comparison between the two groups. The experiment was repeated 3 times independently.

To explore the molecular mechanism of DNMT3B-mediated miR-29b in the development of OA, the expression of DNMT3B in OA cartilage tissues and normal cartilage tissues was determined by the RT-qPCR and Western blot analyses. Our results showed that the expression of DNMT3B in cartilage tissues of patients with OA was lower than that of normal cartilage tissues (Figure 1A, 1B). The results of RT-qPCR displayed that the expression of miR-29b in cartilage tissues of patients with OA was higher than that in normal cartilage tissues (Figure 1C). Pearson’s correlation analysis suggested that DNMT3B expression was negatively correlated with miR-29b expression (Figure 1D).

### DNMT3B inhibits miR-29b by enhancing DNA methylation in the miR-29b promoter region

**Figure 2:**
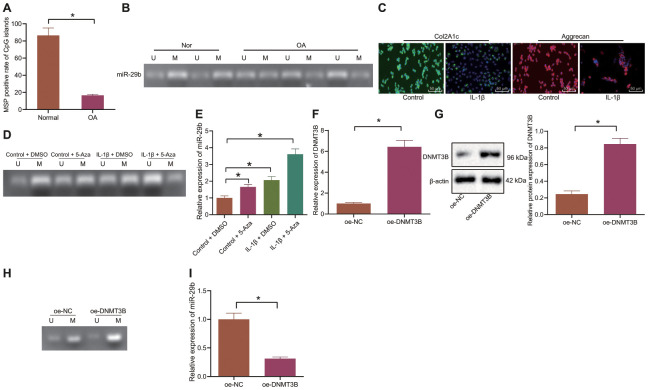
**DNMT3B suppresses the expression of miR-29b by enhancing DNA methylation in the miR-29b promoter region.** (**A**, **B**) The methylation level of miR-29b in cartilage tissues from OA patients (n = 46) and normal cartilage tissues (n = 46) detected by MSP (Nor, normal cartilage tissue; OA, cartilage tissues in patients with OA; U; unmethylated status; M, methylated status). (**C**) Immunofluorescence staining of Col2A1 and Aggrecan in chondrocytes (× 200, scale bar = 50 μm). (**D**) The methylation level of miR-29b in chondrocytes under IL-1β and 5-Aza treatment by MSP (U; unmethylated status; M, methylated status). (**E**) The expression of miR-29b in chondrocytes measured by RT-qPCR. (**F**) The mRNA expression of DNMT3B in chondrocytes measured by RT-qPCR. (**G**) The protein expression of DNMT3B in chondrocytes normalized to β-actin measured by Western blot analysis. (**H**) The methylation level of miR-29b in response to overexpression of DNMT3B determined by MSP. (**I**) The expression of miR-29b in response to overexpression of DNMT3B determined by RT-qPCR. ^*^ *p* < 0.05. Statistical data were measurement data and described as the mean ± standard deviation. The independent sample *t*-test was conducted for comparison between the two groups. The one-way ANOVA was used for comparison among multiple groups followed by Tukey’s post hoc test. The experiment was repeated 3 times independently.

The methylation of CpG island in the miR-29b promoter region of cartilage tissues in patients with OA and matched controls was detected by MSP. The results showed that the hypermethylated rate of CpG island in the miR-29b promoter region of OA cartilage tissues (19.1%) was notably lower than that in normal cartilage tissues (83.3%) (*p* < 0.0001; Figure 2A) whereas the methylation level in the OA cartilage tissues was lower than in the normal cartilage tissues (Figure 2B).

Following after, we isolated the normal chondrocytes which were subsequently treated with IL-1β to simulate the setting of OA chondrocytes. The cartilage markers, Col2A1 and Aggrecan were probed by the immunofluorescence staining and the results showed that the percentages of Col2A1 and Aggrecan positive cells treated with IL-1β were notably reduced (Figure 2C), implying the successful inducement of OA chondrocytes. Then, normal chondrocytes and IL-1β-treated chondrocytes were exposed to 5-Aza-2'-deoxycytidine (5-Aza) reagent (the DNA methylase inhibitor). The methylation of miR-29b was detected by MSP and its expression was determined by the RT-qPCR. Our results demonstrated that IL-1β treatment attenuated the methylation of miR-29b, thus upregulating its expression. Additionally, 5-Aza treatment also weakened the methylation of miR-29b and upregulated its expression (Figure 2D, 2E). The oe-DNMT3B plasmid was transfected into cells and RT-qPCR and Western blot analyses confirmed the successful transfection (Figure 2F, 2G). In the cells overexpressing DNMT3B, the methylation of miR-29b was promoted and its expression was remarkably reduced (Figure 2H, 2I). Collectively, these data indicated that DNMT3B could inhibit the expression of miR-29b by enhancing DNA methylation in the miR-29b promoter region.

### DNMT3B elevates PTHLH expression by inhibiting miR-29b in IL-1β-treated chondrocytes

**Figure 3:**
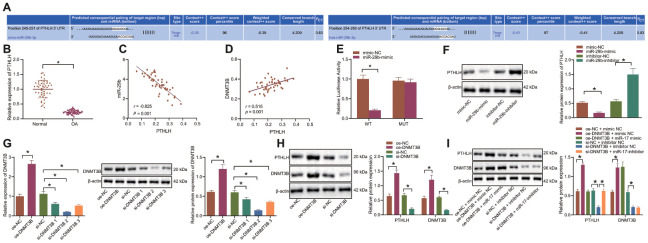
**DNMT3B upregulates PTHLH through the downregulation of miR-29b in IL-1β-treated chondrocytes.** (**A**) Prediction of the binding site between miR-29b and PTHLH available at the Targetscan database. (**B**) The mRNA expression of PTHLH in OA cartilage tissues (n = 46) and normal cartilage tissues (n = 46) by RT-qPCR. (**C**) Pearson’s analysis of the correlation between PTHLH and miR-29b expression. (**D**) Pearson’s analysis of the correlation between PTHLH and DNMT3B expression. (**E**) Luciferase activity of cells co-transfected with PTHLH-WT or PTHLH-MUT and miR-29b mimic or mimic-NC. The firefly luciferase activity was normalized to Renilla luciferase activity. (**F**) The protein expression of PTHLH in response to transfection with miR-29b mimic or inhibitor normalized to β-actin determined by Western blot analysis. (**G**) The expression of DNMT3B after transfection with oe-DNMT3B or si-DNMT3B measured by RT-qPCR or Western blot analysis. (**H**) The protein expression of PTHLH in response to transfection with oe-DNMT3B or si-DNMT3B normalized to β-actin determined by Western blot analysis. (**I**) The protein expression of PTHLH in response to co-transfection with oe-DNMT3B and miR-29b mimic or si-DNMT3B and miR-29b inhibitor normalized to β-actin determined by Western blot analysis. ^*^ *p* < 0.05. Statistical data were measurement data and described as the mean ± standard deviation. The independent sample *t*-test was conducted for comparison between the two groups. The one-way ANOVA was used for comparison among multiple groups followed by Tukey’s post hoc test. The experiment was repeated 3 times independently.

The miRNA target prediction program (http://www.targetscan.org/vert_71/) indicated that PTHLH was a putative target of miR-29b (Figure 3A). The expression of PTHLH in cartilage tissues from OA patients and matched controls was determined by the RT-qPCR. Our results showed that the expression of PTHLH was poor in cartilage tissues of patients with OA (Figure 3B). Moreover, Pearson’s correlation analyses suggested that PTHLH was negatively correlated with miR-29b expression (Figure 3C) and positively correlated with DNMT3B expression (Figure 3D). The luciferase activity of PTHLH-WT was inhibited by miR-29b mimic whereas the luciferase activity of PTHLH-MUT remained unchanged (Figure 3E), as revealed by the dual-luciferase reporter gene assay. Additionally, miR-29b was found to inhibit the protein expression of the PTHLH in chondrocytes while its inhibitor increased the protein expression of PTHLH (Figure 3F). The DNMT3B expression was then altered in the chondrocytes by overexpression/silencing of DNMT3B, and RT-qPCR and Western blot analyses confirmed the successful transfection. The siRNA targeting DNMT3B for better silencing was selected (Figure 3G). Western blot analysis revealed that DNMT3B upregulated the protein expression of PTHLH and silencing of DNMT3B resulted in the downregulation of PTHLH protein expression (Figure 3H). To further investigate whether miR-29b was indispensable in the regulation of DNMT3B in PTHLH, chondrocytes were co-transfected with oe-DNMT3B and miR-29b mimic or si-DNMT3B and miR-29b inhibitor. As shown in Figure 3I, DNMT3B overexpression induced the upregulation of PTHLH protein expression, while miR-29b mimic reversed this upregulation. Similarly, the expression of PTHLH was downregulated by the silencing of DNMT3B while its reduction was rescued by miR-29b inhibitor. Based on these aforementioned results, our data supported the conclusion that DNMT3B could promote PTHLH expression by inhibiting miR-29b in IL-1β-treated chondrocytes.

### PTHLH impedes the apoptosis of IL-1β-treated chondrocytes by elevating CDK4

**Figure 4:**
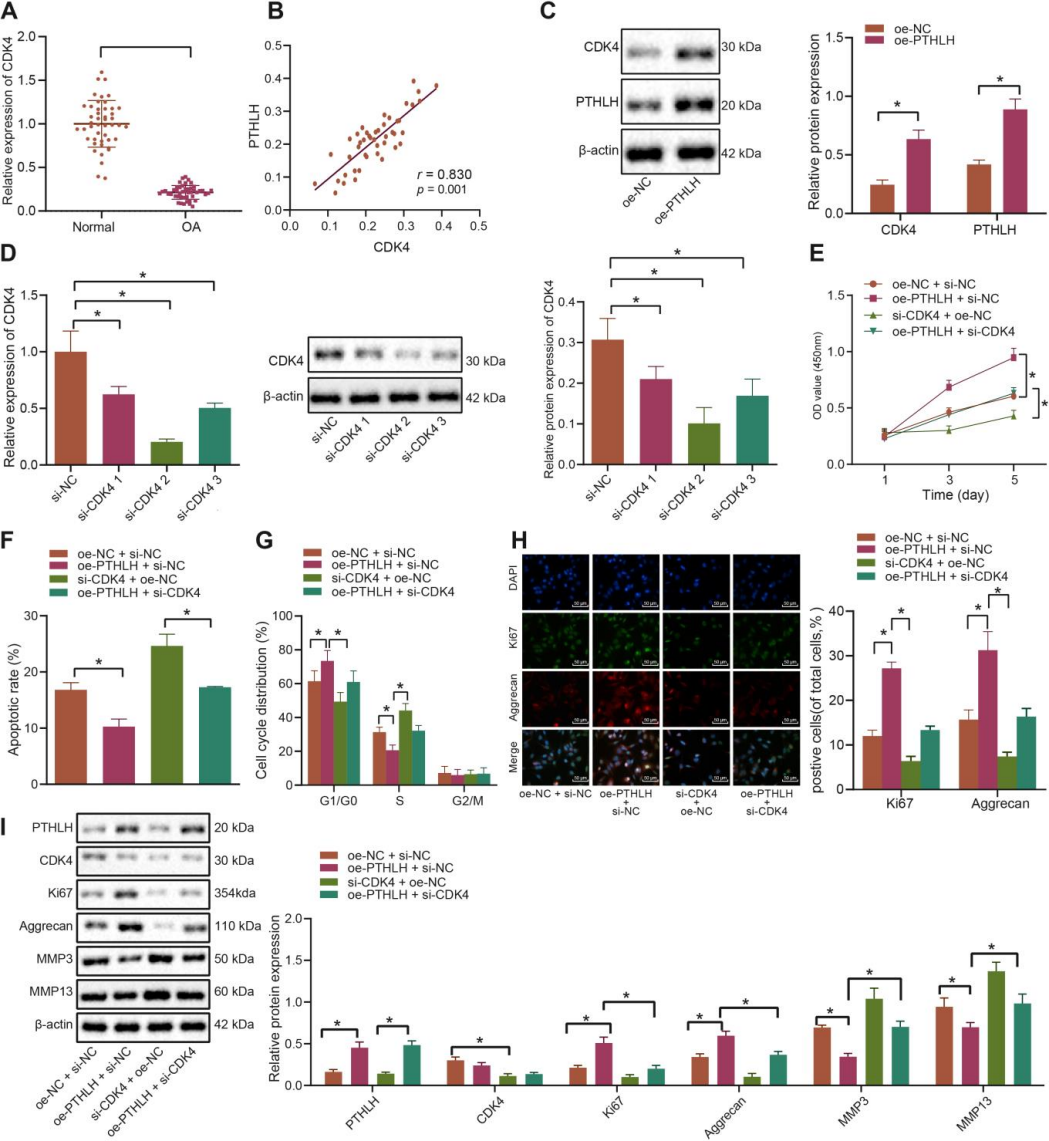
**PTHLH decelerates the apoptosis of IL-1β-treated chondrocytes by enhancing CDK4.** (**A**) The mRNA expression of CDK4 in OA cartilage tissues (n = 46) and normal cartilage tissues (n = 46) determined by RT-qPCR. (**B**) Pearson’s analysis of the correlation between PTHLH and CDK4 expression. (**C**) The protein expression of CDK4 in chondrocytes in response to overexpression of PTHLH normalized to β-actin measured by Western blot analysis. (**D**) The expression of CDK4 after siRNA transfection measured by RT-qPCR and Western blot analysis (normalized to β-actin). In panel (**E**–**I**) IL-1β-treated chondrocytes were co-transfected with oe-PTHLH/oe-NC and si-CDK4/si-NC. (**E**) Viability of IL-1β-treated chondrocytes evaluated by MTT assay. (**F**) Apoptosis of IL-1β-treated chondrocytes assessed by flow cytometry. (**G**) Cell cycle distribution of IL-1β-treated chondrocytes assessed by flow cytometry. (**H**) Immunofluorescence staining of Ki67 and Aggrecan in IL-1β-treated chondrocytes (× 200, scale bar = 50 μm). (**I**) The protein expression of Ki67, Aggrecan, MMP3, MMP13 in IL-1β-treated chondrocytes normalized to β-actin measured by Western blot analysis. ^*^ *p* < 0.05. Statistical data were measurement data and described as the mean ± standard deviation. The independent sample *t*-test was conducted for comparison between the two groups. The one-way ANOVA was used for comparison among multiple groups followed by Tukey’s post hoc test. The experiment was repeated 3 times independently.

Thereafter, the potential regulatory role of PTHLH in the development of OA was investigated. RT-qPCR was performed to determine the expression of CDK4 in the cartilage tissues of OA patients and controls, and our results showed that CDK4 was poorly expressed in the cartilage tissues of patients with OA (Figure 4A). Furthermore, CDK4 was shown to be positively correlated with PTHLH expression (Figure 4B). The oe-PTHLH plasmid was introduced into chondrocytes and the quantification analysis results showed that PTHLH overexpression elevated the protein expression of CDK4 (Figure 4C). For CDK4 knockdown experiments, the siRNA targeting CDK4 with the best silencing efficacy was determined by RT-qPCR and Western blot analyses (Figure 4D), respectively. The *in-vitro* viability of IL-1β-treated chondrocytes assessed by MTT assay was significantly enhanced after overexpression of PTHLH (*p* < 0.05), however, this enhancement was blocked by co-transfection with si-CDK4 (*p* < 0.05; Figure 4E). Additionally, overexpression of PTHLH decreased the number of apoptotic IL-1β-treated chondrocytes as demonstrated by flow cytometry (*p* < 0.05), while the number of apoptotic cells was remarkably increased by CDK4 silencing (*p* < 0.05); on the other hand, the inhibition of apoptosis by oe-PTHLH was partially counteracted by si-CDK4 (Figure 4F). Consistently, flow cytometric analysis also demonstrated that the silencing of CDK4 induced G1 cell cycle arrest and notably decreased the number of S-phase cells in IL-1β-treated chondrocytes (*p* < 0.05). On contrary, overexpression of PTHLH led to increased number of cells in the S-phase, indicative of cell cycle progression (Figure 4G). The expression of Ki67 and Aggrecan in IL-1β-treated chondrocytes was detected by the immunofluorescence, and the percentages of Ki67 and Aggrecan positive cells were increased by the overexpression of PTHLH; however, this increase was alleviated by additional transfection with si-CDK4 (Figure 4H). Consistently, results from Western blot analysis also exhibited that the protein expression of Ki67 and Aggrecan was elevated, while the expression of ECM markers MMP3 and MMP13 was downregulated in IL-1β-treated chondrocytes by overexpression of PTHLH. However, the silencing of CDK4 partially inhibited the increase of Ki67 and Aggrecan and the decline of ECM markers which were induced by PTHLH overexpression (Figure 4I). Collectively, these aforementioned-results suggested that PTHLH could accelerate the viability of IL-1β-treated chondrocytes and hinder their apoptosis in OA in a CDK4-dependent manner.

### DNMT3B promotes RUNX2 ubiquitination induced by CDK4

**Figure 5:**
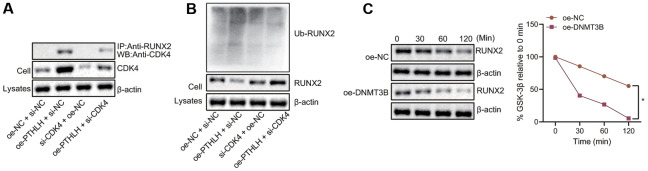
**DNMT3B promotes RUNX2 ubiquitination and proteasome degradation in a CDK4-dependent manner.** (**A**) The interaction between endogenous RUNX2 and CDK4 in chondrocytes detected by Co-IP assay. (**B**) The expression of ubiquitinated RUNX2 protein normalized to β-actin measured by Western blot assay. (**C**) The degradation of RUNX 2 in chondrocytes treated with CHX. ^*^ *p* < 0.05. Statistical data were measurement data and described as the mean ± standard deviation. The repeated-measures ANOVA was applied for the comparison of data at different time points followed by Bonferroni’s post hoc test. The experiment was repeated 3 times independently.

To investigate whether the CDK4-mediated RUNX2 ubiquitination was regulated by the DNMT3B in chondrocytes, Co-IP was performed using the anti-RUNX2 antibody and the CDK4-mediated RUNX2 ubiquitination was detected by the Western blot assay. In Input samples, the expression of CDK4 in chondrocytes was increased upon overexpression of DNMT3B and enhancement of interaction between CDK4 and RUNX2 was observed (Figure 5A). Meanwhile, the expression of the ubiquitinated RUNX2 protein in the chondrocytes was elevated by overexpression of DNMT3B, while the downregulation of CDK4 inhibited the ubiquitination of RUNX2 induced by DNMT3B in the chondrocytes (Figure 5B). Following after, cells were treated with protein synthesis inhibitor cycloheximide (CHX) to determine the effect of DNMT3B on RUNX2 protein degradation. After 2 hours of CHX treatment, the expression of RUNX2 protein was gradually decreased whereas in chondrocytes overexpressing DNMT3B, the degradation of RUNX2 protein was significantly faster (Figure 5C). Thus, these results suggested that DNMT3B could induce the ubiquitination of RUNX2 protein by upregulating the CDK4.

### DNMT3B enhances the apoptosis of IL-1β-treated chondrocytes by downregulation of RUNX2

**Figure 6:**
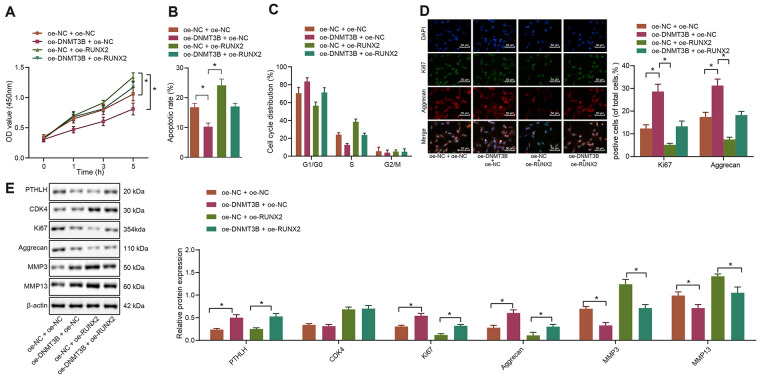
**DNMT3B modulates the functions of IL-1β-treated chondrocytes through the downregulation of RUNX2.** IL-1β-treated chondrocytes were co-transfected with oe-DNMT3B/oe-NC and oe-RUNX2/oe-NC. (**A**) The viability of IL-1β-treated chondrocytes evaluated by MTT assay. (**B**) The apoptosis of IL-1β-treated chondrocytes assessed by flow cytometry. (**C**) The cell cycle distribution of IL-1β-treated chondrocytes assessed by flow cytometry. (**D**) Immunofluorescence staining of Ki67 and Aggrecan in IL-1β-treated chondrocytes (× 200, scale bar = 50 μm). (**E**) The protein expression of Ki67, Aggrecan, MMP3, MMP13 in IL-1β-treated chondrocytes normalized to β-actin measured by Western blot analysis. ^*^ *p* < 0.05. Statistical data were measurement data and described as the mean ± standard deviation. The one-way analysis of variance was used for comparison among multiple groups, followed by Tukey’s post hoc test. The repeated-measures ANOVA was applied for the comparison of data at different time points, followed by Bonferroni’s post hoc test. The experiment was repeated 3 times independently.

To further investigate whether DNMT3B was involved in the regulation of RUNX2 in IL-1β-treated chondrocytes, both RUNX2 and DNMT3B were overexpressed in chondrocytes either in combination or alone. The MTT assay demonstrated that overexpression of DNMT3B remarkably promoted the *in-vitro* viability of IL-1β-treated chondrocytes (*p* < 0.05) while overexpression of RUNX2 noticeably inhibited their viability (*p* < 0.05). Furthermore, overexpression of RUNX2 neutralized the enhancement of IL-1β-treated chondrocyte viability caused by DNMT3B overexpression (Figure 6A). Additionally, flow cytometric data suggested that overexpression of DNMT3B inhibited the apoptosis of IL-1β-treated chondrocytes while overexpression of RUNX2 enhanced their apoptosis (*p* < 0.05). Overexpression of RUNX2 attenuated the suppression of apoptosis induced by DNMT3B overexpression (Figure 6B). Meanwhile, overexpression of RUNX2 induced G1 cell cycle arrest and decreased the cells at S phase in IL-1β-treated chondrocytes (*p* < 0.05). The upregulation of DNMT3B, conversely, resulted in increased cells in S-phase and promoted cell cycle progression (Figure 6C). The results from immunofluorescence assay further revealed that the percentages of Ki67 and Aggrecan positive cells were increased in IL-1β-treated chondrocytes by overexpression of DNMT3B but were reduced by overexpression of RUNX2 (Figure 6D). Besides, Western blot analysis exhibited that the protein expression of Ki67 and Aggrecan was increased while the expression of ECM markers MMP3 and MMP13 was downregulated in IL-1β-treated chondrocytes following oe-DNMT3B delivery. Moreover, overexpression of RUNX2 reversed the increase of Ki67 and Aggrecan and the downregulation of ECM markers that were induced by DNMT3B (Figure 6E). Hence, these above-described results collectively indicated that RUNX2 could alleviate the effects of DNMT3B on IL-1β-treated chondrocytes.

### DNMT3B inhibits the progression of OA through RUNX2 downregulation *in vivo*

**Figure 7:**
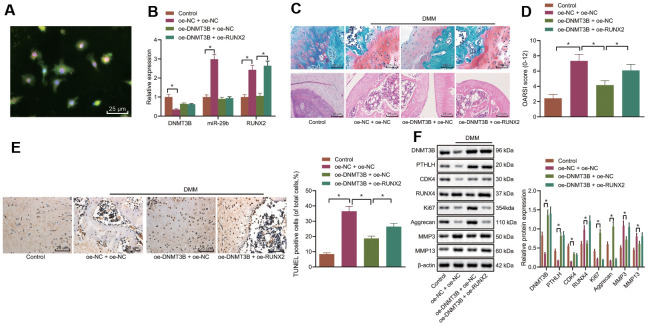
**DNMT3B inhibits OA progression in the mouse model of OA.** A mouse model of OA was induced by DMM and treated with lentivirus vector harboring oe-DNMT3B and/or oe-RUNX2. (**A**) Co-localization of DNMT3B and collagen II in cartilage tissues of mice detected by immunofluorescence (× 400, scale bar = 25 μm). (**B**) The miR-29b expression and the mRNA expression of DNMT3B and RUNX2 in cartilage tissues of mice determined by RT-qPCR. (**C**) Histological analyses including Safranin O staining and HE staining and microscopic observation of cartilage destruction at 8 weeks post-operation (lesions of articular cartilage were indicated by black arrows, × 400, scale bar = 25 μm). (**D**) OARIS scores of articular cartilage. (**E**) Representative images (× 400, scale bar = 25 μm) and quantitation of TUNEL positive apoptotic cells in the cartilage tissues. (**F**) The protein expression of CDK4, Ki67, MMP3, and MMP13 in cartilage tissues normalized to β-actin measured by Western blot analysis. ^*^ *p* < 0.05. Statistical data were measurement data and described as the mean ± standard deviation. The one-way ANOVA was used for comparison among multiple groups, followed by Tukey’s post hoc test. The experiment was repeated 3 times independently.

The mouse model of OA was induced by the DMM and lentivirus vector harboring oe-DNMT3B and/or oe-RUNX2 were injected into mice intraarticularly. After 8 weeks, the mice were euthanized and the cartilage tissues were collected from their hind limbs. Immunofluorescence was performed to detect the co-localization of DNMT3B and collagen II in the cartilage tissues (Figure 7A), and results revealed that DNMT3B and collagen II were both localized in the cartilage tissues. The expression of DNMT3B, miR-29b, and RUNX2 in the collected cartilage tissues was determined by the RT-qPCR (Figure 7B), followed by histological analyses. The results of Safranin O and HE staining (Figure 7C) showed that the surface of the knee cartilage (red) of the sham-operated mice was smooth. In DMM-operated mice, severe destruction, erosion, and lesions of articular cartilage, a remarkable reduction of chondrocytes, as well as the enormous loss of proteoglycan, were observed. However, the upregulation of DNMT3B ameliorated the loss of chondrocytes and proteoglycan in the DMM-operated mice. Nevertheless, oe-RUNX2 reversed the ameliorative effect of oe-DNMT3B on the loss of chondrocytes and proteoglycan. Meanwhile, OARSI scoring presented consistent results with histological analysis (Figure 7D). OARSI score of the DMM-operated mice was markedly increased compared with the sham-operated mice, while overexpression of DNMT3B reduced the OARSI score of the DMM-operated mice. The cell apoptosis in cartilage tissues shown by TUNEL staining was enhanced in the DMM-operated mice, which was inhibited by the upregulation of DNMT3B whereas RUNX2 overexpression partially counteracted the anti-apoptotic effect of DNMT3B (Figure 7E). Results from Western blot analysis demonstrated that overexpression of DNMT3B resulted in reduced expression of RUNX2, MMP3, and MMP13 in cartilage tissues, and potentiated expression of PTHLH, CDK4, Ki67, and Aggrecan. On the contrary, RUNX2 overexpression reversed the changes in the above-mentioned proteins caused by DNMT3B overexpression (Figure 7F). Hence, these data suggested that DNMT3B exerted an anti-apoptotic function *in-vivo* by reducing RUNX2, which was consistent with our *in-vitro* findings.

## DISCUSSION

Based on the previous evidence, the apoptosis of massive chondrocytes that results in cartilage degeneration and subchondral bone thickening could be responsible for the development and progression of OA [14, 15]. The present study demonstrated that DNMT3B could methylate miR-29b and was involved in the regulation of chondrocyte proliferation and apoptosis, as well as ECM degradation. Besides, miR-29b was identified to directly bind to the 3’UTR of PTHLH. Moreover, by using gain-of-function and loss-of-function approaches *in vitro*, we experimentally demonstrated that DNMT3B blocked the ECM degradation and inhibited the apoptosis of chondrocytes by impairing the miR-29b-dependent downregulation of PTHLH and enhancing the CDK4-induced RUNX2 ubiquitination.

Initially, we corroborated the upregulation of miR-29b and downregulation of DNMT3B and PTHLH in patients with OA. Accordingly, a recent study has demonstrated that several epigenetic regulators were associated with bone or cartilage regeneration therapeutics, such as DNA methyltransferases including DNMT1, DNMT3A, and DNMT3B [16]. For instance, targeted DNMT3B knockout in murine articular chondrocytes has been demonstrated to contribute to an early-onset and progressive postnatal OA-like pathology [17]. However, the local delivery of DNMT3B has been shown to partially alleviate the OA-induced cartilage degradation [7]. Nevertheless, DNA methylation is a major mechanism of gene expression modulation in a diversity of organisms. Whilst DNMT3B has been indicated to binds to the Abat in the promoter region to induce the methylation of a conserved CpG sequence, consequently reducing the Abat expression and potentially protecting against OA induced by injury [18]. Consistently, in the present study, miR-29b was found to be conversely correlated with DNMT3B expression in 46 clinical cartilage tissues of patients with OA. Moreover, we found that DNMT3B could inhibit the expression of miR-29b by enhancing the DNA methylation in the miR-29b promoter region. In consent with our findings, miR-29b has been reported to have a negative correlation with DNMT3B in Burkitt lymphoma cells, indicating that miR-29b overexpression could decrease the DNMT3B expression, and in turn, miR-29b was inhibited by the DNMT3B in a DNA methylation-dependent manner [19]. Intriguingly, miR-29b confers either supportive or possibly unfavorable effects on OA cartilage regeneration by mediating the chondrogenic differentiation of bone marrow-derived mesenchymal stem cells [20]. Moreover, it has been illustrated that miR-29b could facilitate hypertrophy in chondrocytes derived from murine mesenchymal stem cells [21]. In this study, DNMT3B was suggested to inhibit miR-29b, thereby impeding the apoptosis of chondrocytes. Thus, it indicated that the inhibition of chondrocyte apoptosis was likely attributed to the methylation of miR-29b. In addition to DNMT3B, DNMT3A has also been indicated to play a crucial role in the mediation of miR-29b in a positive feedback loop [22] which highly suggested the requirement of further investigation on whether DNMT3A possess similar function and mechanisms in the progression of OA.

Meanwhile, our study identified that the PTHLH was a target of miR-29b and PTHLH overexpression resulted in enhanced CDK4, thus promoting the CDK4-induced RUNX2 ubiquitination. The PTHLH is also known as PTHrP and its downregulation by miR-195 may accelerate the onset and progression of OA [23]. In the present study, overexpression of PTHLH contributed to an elevated chondrocyte proliferation while reducing apoptotic chondrocytes. In consent with our findings, a previously reported study has indicated that PTHLH protects chondrocyte from premature hypertrophy by inducing the CDK1-mediated RUNX2 ubiquitylation and proteasomal degradation [24]. However, the silencing of CDK4 counteracted the stimulative effect of PTHLH on chondrocyte proliferation. Consistently, it has been reported that increased CDK4 can strengthen the IL-1β-induced chondrocyte proliferation through the acceleration of G1/S transition [25]. The RUNX2 has been documented to be upregulated in chondrocytes in OA cartilage and the loss of RUNX2 in chondrocytes decelerates OA progression since Indian hedgehog (Ihh) potentiates the PTHLH, which further inhibits the RUNX2 and chondrocyte maturation, generating a negative feedback loop for chondrocyte maturation [26]. Additionally, gene expression of PTHrP and Ihh has been reported to be diminished in the articular cartilage as detected from a surgically induced rat model of OA [27]. Given the potential role of Ihh in OA involving PTHrP, future research focus will be shifted to the impact of miR-29b, DNMT3B or CDK4 on Ihh. Notably, our data supported the previous findings that the PTHLH overexpression led to the CDK4 enhancement whereas CDK4 induced the RUNX2 protein ubiquitination in chondrocytes, suggesting that the specific interaction between PTHLH and RUNX2 warrants further exploration.

Of note, the present study demonstrated that DNMT3B overexpression resulted in significant upregulation of Ki67 and Aggrecan expression and downregulation of ECM markers, MMP3 and MMP13, in chondrocytes and cartilage tissues extracted from DMM-operated mice, respectively. On the contrary, the addition of RUNX2 reversed the aforementioned regulatory effects of DNMT3B, indicating the positive role of RUNX2 in the modulation of ECM degradation. Consistently, the cell proliferation marker (Ki67) was suppressed by IL-1β treatment in the OA chondrocytes, indicating the inhibition of proliferation, and was further observed to be involved in the chondrogenic ECM degradation and OA progression [28]. Concordantly, the elevated expression of Aggrecan (a main cartilage matrix component) while reduced expression of MMP13 (a cartilage degrading enzyme) were reported in primary human OA chondrocytes after delivery of fibroblast growth factor receptor antagonists featured with promising beneficial action [29]. Whilst downregulation of MMP3 and MMP13 has been reported to be indicative of the chondroprotective action of Icariin in SW1353 chondrosarcoma cells stimulated with IL-1β [30]. In consistent with the present study, a significantly enhanced expression of MMP13 was found in the middle and superficial zones of the cartilage in mice after DMM surgery, suggesting that chondrocyte hypertrophy was enhanced [31]. Moreover, Tetsunaga et al. have also suggested that overexpression of the RUNX2 upregulates the expression of MMP13 in chondrocyte-like cells [32]. Accordingly, the results from loss-of-function of DNMT3B have demonstrated to elevate the expression of factors related to chondrocyte catabolism and terminal hypertrophic differentiation, such as MMP13 and RUNX2 [17]. Another study has revealed that the deletion of DNMT3B in chondrocytes constrains the hypertrophic differentiation of chondrocytes and matrix mineralization *in-vitro* [33].

Collectively, this study proposes that DNMT3B downregulated the miR-29b by promoting the DNA methylation in the miR-29b promoter region to upregulate the PTHLH, the target gene of miR-29b. Thus, the CDK4-mediated RUNX2 ubiquitination was enhanced to reduce the RUNX2 expression, by which chondrocyte apoptosis and extracellular matrix degradation were suppressed, curbing OA progression. These findings may suggest a possible insight and a better understanding of the pathological mechanisms underlying OA development. Our results were consistent with a recent study indicating CDK6 as a direct target of miR-29b in lung cancer [34]. Thus, the interaction between CDK4 and miR-29b free of PTHLH regulation would be the focus of our future researches.

## MATERIALS AND METHODS

### Human articular cartilage tissue collection

The OA articular cartilages were obtained from 46 OA patients (aged 40 - 65 years) who received total knee arthroplasty. The 46 normal articular cartilages were isolated from patients with acute injuries or amputations (aged 42 - 60 years). All cases were collected from the Orthopedic Department of The Second Xiangya Hospital, Central South University from January 2015 to March 2019.

### Primary culture of chondrocytes

Under aseptic conditions, the cartilage tissues were repeatedly washed with phosphate-buffered saline (PBS) to remove the coagulation and appendages and cut into 1 mm^3^ blocks. Subsequently, the tissues were detached with 0.05% type II collagenase (Sigma-Aldrich Chemical Company, St Louis, MO, USA) for 30 minutes, centrifuged at 1000 r/min with supernatant discarded, followed by detachment with the mixture of 0.1% type II collagenase and 0.25% trypsin (Sigma-Aldrich) at 37°C for 60-100 minutes. Afterward, the cells were dispersed into cell suspension in Dulbecco’s modified Eagle’s medium (Gibco, Carlsbad, CA, USA), which was supplemented with 10% fetal bovine serum (Gibco), 100 units/mL penicillin, and 100 units/mL streptomycin. Chondrocytes (4 × 10^5^ cells/mL) were grown on culture plates and incubated at 37°C under 5% CO_2_. Chondrocytes at passage 2 - 3 were used for the subsequent experiments. The primary chondrocytes were stimulated with 10 ng/mL interleukin-1 beta (IL-1β) for 12 hours to induce inflammation *in vitro*.

### Cell treatment

Small interfering RNA (siRNA) targeting DNMT3B (si-DNMT3B), siRNA targeting CDK4 (si-CDK4), DNMT3B overexpression plasmid (oe-DNMT3B), oe-PTHLH, oe-RUNX2, miR-29b mimic, miR-29b inhibitor, nonsense siRNA for negative control (si-NC), nonsense RNA sequence (mimic NC and inhibitor NC) were employed for cell transfection with empty vector (oe-NC) serving as the control. The cells were transfected with the aforementioned siRNA i.e., miRNA mimic, miRNA inhibitor, plasmids and NC plasmids in compliance with the specifications of the Lipofectamine 2000 (Invitrogen, Carlsbad, CA, USA).

### Lentivirus construction

The overexpressed DNMT3B sequence and RUNX2 sequence provided by the Millipore (Billerica, MA, USA) were inserted to the pLKO-Puro vector (Millipore), respectively. The above plasmids were co-transduced with pPAX2 and pMD2.G (Adgene, Watertown, MA, USA) into human embryonic kidney 293T cells.

### RNA isolation and quantification

Total RNA was extracted from cartilage tissues and cells according to the manufacturer’s protocols of the RNeasy Mini Kit (Qiagen, Valencia, CA, USA). The cDNA was obtained by reverse transcription (RT) using an RT kit (RR047A, Takara Holdings Inc., Kyoto, Japan) and miRNA First Strand cDNA Synthesis (B532451-0020, Shanghai Sangon Biological Engineering Technology and Services Co., Ltd., Shanghai, China). The real-time quantitative polymerase chain reaction (qPCR) was conducted using the SYBR^®^ Premix Ex TaqTM II (PerfectReal Time) kit (DRR081, Takara) in a real-time fluorescence qPCR instrument (ABI 7500, ABI, Foster City, CA, USA). The real-time PCR primers are indicated in Table 1. Relative quantification analysis was performed using the comparative Ct method.

**Table 1:** Primer sequences for RT-qPCR.

**Gene**	**Primer sequences**
DNMT3B	Forward: 5'-CTAAGAGCGTCAGTACCCCATC-3'
	Reverse: 5'-CACGAGGTCACCTATTCCAAA-3'
miR-29b	Forward: 5'-ACACTCCAGCTGGGTAGCACCATTTGAAATC-3'
	Reverse: 5'-TGGTGTCGTGGAGTCG-3'
PTHLH	Forward: 5'-AGAGGAACTGCGACGAACA-3'
	Reverse: 5'-GGATTGATTTGCCCTTGTC-3'
CDK4	Forward: 5'-ATGTTGTCCGGCTGATGG-3'
	Reverse: 5'-CACCAGGGTTACCTTGATCTCC-3'
RUNX2	Forward: 5'-GACTGTGGTTACCGTCATGGC-3'
	Reverse: 5'-ACTTGGTTTTTCATAACAGCGGA-3'
U6	Forward: 5'-GCTTCGGCAGCACATATAC-3'
	Reverse: 5'-AACGCTTCACGAATTTGCGT-3'
GAPDH	Forward: 5'-AATGGATTTGGACGCATTGGT-3'
	Reverse: 5'-TTTGCACTGGTACGTGTTGAT-3'

Note: RT-qPCR, reverse transcription quantitative polymerase chain reaction; DNMT3B, DNA methyltransferase 3B; miR-29b, microRNA-29b; PTHLH, parathyroid hormone-like hormone; CDK4, cyclin-dependent kinase 4; RUNX2, runt-related gene 2; GAPDH, glyceraldehyde-3-phosphate dehydrogenase.

### Western blot analysis

The total protein in tissue and cells was extracted using radio-immunoprecipitation assay lysis buffer containing phenylmethanesulfonyl fluoride (R0010, Solarbio Science and Technology Co., Ltd., Beijing, China) and incubated on ice for 30 minutes. A bicinchoninic acid assay Protein Assay Kit (23225, Pierce, Waltham, MA, USA) was used to determine the protein concentration. The extracted protein samples were separated by 10% sodium dodecyl sulfate-polyacrylamide gel electrophoresis (P0012A, Beyotime, Shanghai, China) and subsequently transferred onto a polyvinylidene fluoride membrane (ISEQ00010, Millipore). After blocking with 5% non-fat milk in Tris-buffered saline tween for 2 h, the membranes were incubated overnight at 4°C with primary antibodies as follows: DNMT3B (1:1000, ab122932), PTHLH (1:1000, ab125700), CDK4 (1:1000, ab137675), RUNX2 (1:1000, ab76956), Ki67 (1:1000, ab15580), Aggrecan (1:1000, ab36861), matrix metalloproteinase3 (MMP3) (1:1000, ab52915), MMP13 (1:1000, ab39012) and β-actin (1:1000, ab8227). All antibodies were purchased from Abcam Inc. (Cambridge, UK). Then, the membrane was incubated with horseradish peroxidase-coupled goat anti-rabbit secondary antibody to immunoglobulin G (IgG) (1:5000, Beijing Zhongshan Biotechnology Co., Ltd., Beijing, China). The signals of the bands were visualized by enhanced chemiluminescence. Quantity One v4.6.2 software was employed to quantify the expression of proteins with β-actin as the loading control.

### Cell viability assessment

The cells were seeded on 96 well plates at a cell density of 3 × 10^3^ cells/well. After treating with 10 ng/mL IL-1β for 12 h, 10 μL 3-(4,5-Dimethylthiazol-2-Yl)-2,5-Diphenyltetrazolium Bromide (MTT) solution was added to each well for 4-hours incubation at 37°C. Optical density was recorded using a microplate reader at 570 nm.

### Flow cytometry

Chondrocytes were fixed with pre-chilled anhydrous ethanol overnight at 4°C, washed with PBS, and centrifuged at 2000 r/min. A total of 500 μL 1 × fluorescent-activated cell sorter buffer containing 0.1% bovine serum albumin (BSA), PBS, 0.01% NaN_3,_ and 2.5 mL ribonuclease A (10 mg/mL) was added to chondrocytes. After complete mixing, the cells were reacted with 25 μL propidium iodide (PI; 1 mg/mL; Beyotime) for 15 minutes in the dark. The cell cycle was detected by a flow cytometer (FACS CantoII; Becton, Dickinson and Company, Franklin Lakes, NJ, USA).

After 48 hours of transfection, chondrocytes were trypsinized in the absence of ethylenediaminetetraacetic acid and centrifuged. The apoptotic rate of chondrocytes was measured by using an Annexin-V-fluorescein isothiocyanate (FITC)/PI apoptosis detection kit (556547, Shanghai Soljia technology Co., Ltd., Shanghai, China). Annexin-V-FITC, PI, and N-2-Hydroxyethylpiperazine-N'-2-ethane sulfonic acid (HEPES) buffer solution were prepared at a ratio of 1:2:50. A total of 1 × 10^6^ cells were resuspended by every 100 μL solution and incubated at room temperature for 15 minutes followed by the addition of 1 mL HEPES buffer. Subsequently, FITC and PI signals were detected at the wavelength of 488 nm with emitting wavelengths of 515 nm and 620 nm, respectively for the detection of cell apoptosis.

### Dual-luciferase reporter gene assay

The relationship between miR-29b and PTHLH was predicted by biological prediction websites and verified by the dual-luciferase reporter gene assay. The PTHLH 3’untranslated region (3’UTR) fragment sequences containing miR-29b binding sites and the mutant sequence based on site-directed mutation were cloned into the psiCheck2 vector, named PTHLH-wild type (WT), and PTHLH-mutant (MUT). Chondrocytes were transfected with PTHLH-WT and PTHLH-MUT, miRNA mimic or mimic NC. Lysates were harvested 48 hours after transfection. Renilla luciferase activities were consecutively measured according to manufacturer’s protocols of the dual-luciferase reporter gene assay (Promega, Madison, WI, USA). The firefly luciferase signal was normalized to the Renilla luciferase signal.

### Immunofluorescence staining

The treated chondrocytes were fixed with 1 mL 4% paraformaldehyde for 25 minutes, permeated with 0.2% Triton X-100 in PBS for 5 - 10 min, and blocked with 5% BSA for 90 minutes at room temperature. Then, the chondrocytes were incubated at 4°C for 24 hours with the primary antibody, rabbit polyclonal antibody against Ki67(1:1000, ab15580, Abcam) and mouse monoclonal antibody against Aggrecan (1:1000, ab3778, Abcam). Subsequently, Alexa Fluor594-conjugated (ab150116, Abcam) or Alexa Fluor488-conjugated (ab150077, Abcam) secondary antibody was diluted at a 1:500 with PBS and incubated with the chondrocytes in the dark for 90 minutes. Ultimately, 4',6-Diamidino-2-Phenylindole (DAPI) solution was added for 10 minutes incubation in the dark at ambient temperature. A confocal laser scanning microscope (Olympus Optical Co., Ltd., Tokyo, Japan) was employed to observe the positively stained cells. The fluorescence intensity was assessed by the ImageJ software 2.1.

### Methylation-specific polymerase chain reaction (MSP)

The methylation level of the miR-29b promoter was detected using a DNA Methylation-Gold^TM^ kit (D5005, Zymo Research, Irvine, CA, USA). The primer sequences for MSP were as follows: methylated (forward: CGTTTTTTAGTATTATTGTTAGTCGT, reverse: AAAACACATATCAACCCCGTC; unmethylated (forward: TGGTTTTTTAGTATTATTGTTAGTTGT, reverse: AAAAACATATCAACCCCATC). The purified DNA was added to CT conversion reagent for denaturation and bisulfate conversion whereas desulphurization and purification were performed by reaction columns. The purified DNA was used for subsequent PCR which was carried out under the following conditions: at 95°C for 10 minutes followed by 35 cycles of 45 seconds at 95°C, at 56°C (methylation)/45°C (unmethylation), and 45 seconds at 72°C. The reaction products were subjected to agarose gel electrophoresis and image analysis for subsequent experiments.

### Co-immunoprecipitation (Co-IP)

The cells were lysed with cell lysis buffer on ice for 30 minutes and centrifuged. The supernatant was incubated overnight at 4°C with antibodies to RUNX2 or antibody to IgG. The pre-treated protein A agarose beads were then incubated with the antibody-protein complex at 4°C for 2-4 hours. The protein precipitated from centrifugation was detected by Western blot analysis using an anti-ubiquitin antibody.

### Terminal deoxynucleotidyl transferase-mediated 2'-deoxyuridine 5'-triphosphate (dUTP) nick-end labeling (TUNEL) staining

After being fixed with 4% paraformaldehyde for 15 minutes, chondrocytes were rinsed three times with PBS and permeabilized with 0.1% Triton-X 100 in PBS for 3 minutes. Subsequently, the apoptotic chondrocytes were stained with TUNEL staining solution and counterstained with DAPI for 10 minutes. The apoptotic chondrocytes were observed under a confocal microscope and the percentage of apoptotic chondrocytes was calculated.

### Inducement of OA in mice

An OA mouse model was developed by DMM in 48 adult C57BL/6 male mice (aged 5 - 6 months, weighing 18 - 20 g) [35]. One week after the DMM surgery, mice received an intra-articular injection of packaged lentivirus, twice a week for 4 weeks. The animals were euthanized 8 weeks later. The hind limbs were fixed in formalin, decalcified in formic acid, embedded in paraffin, and sectioned for histological and immunohistochemical analyses.

### Histological and immunohistochemical analysis

The sections were fixed with ice-cold acetone for 2 minutes at 20°C followed by 30-minutes blocking with 5% normal goat serum (Vector Laboratories, Burlingame, CA, USA) in PBS containing 0.2% Tween-20 at room temperature. Cryosections were stained overnight at 4°C to allow DNMT3B to bind to collagen II for further co-immunofluorescence staining followed by a 1-hour incubation with FITC-labeled goat anti-rabbit (Sigma-Aldrich) and Alexa 488-labeled goat anti-rat (Invitrogen) at room temperature. After analysis of stained sections, images were acquired under a microscope.

The cartilage tissues were sliced into 4-μm tissue sections. The sections were stained with fast green for 5 minutes and stained with Safranin O for 30 seconds. After that, the sections were dehydrated, cleared with xylene and visualized microscopically.

The cartilage tissues were dehydrated, subsequently embedded in paraffin, and cut into 5-μm tissue sections. Then hematoxylin and eosin (HE) staining were applied to evaluate the degeneration of articular cartilage in each knee joint. The degradation change was assessed and analyzed by an Osteoarthritis Research Society International (OARSI) scoring system.

### Statistical analysis

SPSS 21.0 software (IBM Corporation, Armonk, NY, USA) was used to analyze the data. All data in compliance with normal distribution and equal variance were presented as the mean ± standard deviation. Statistical analysis was performed by an unpaired *t*-test for two groups. For data among multiple groups, one-way analysis of variance (ANOVA) was performed followed by Turkey’s post hoc test. The repeated-measures ANOVA was applied for the comparison of data at different time points followed by Bonferroni’s post hoc test. All *p*-values less than 0.05 were considered significant.

### Ethics statement

Informed consent was obtained from each subject. The study was approved by the Human Ethics Committee of The Second Xiangya Hospital, Central South University. The animal protocol of this study was approved by the Ethics Committee of The Second Xiangya Hospital, Central South University. All experimental methods and procedures were carried out by following the approved guidelines.
